# Parameters of the crossing points between center of pressure and center of mass signals are potential markers of postural control efficiency

**DOI:** 10.1371/journal.pone.0219460

**Published:** 2019-07-12

**Authors:** Krzysztof Piotr Michalak, Anna Przekoracka-Krawczyk, Ryszard Naskręcki

**Affiliations:** 1 Laboratory of Vision Science and Optometry, Faculty of Physics, Adam Mickiewicz University, Poznań, Poland; 2 Vision and Neuroscience Laboratory, NanoBioMedical Centre, Adam Mickiewicz University, Poznań, Poland; University of Illinois at Chicago, UNITED STATES

## Abstract

Posturographic signals were recorded for 384 subjects of different ages and with old persons with gait disturbances. Four conditions were used: Eyes Open/Closed vs. Head Normal/Bent Back. ‘Center of Pressure’ (CoP) signals were decomposed into ‘Center of Mass’ (CoM) and the remaining difference between Center of Pressure and Center of Mass (CoPM). The Zero-Crossing points in which the Center of Mass and Center of Pressure paths cross each other have been extracted. Velocity of CoM, velocity of CoPM and acceleration of CoPM in Zero-Crossing points were analyzed to be potential markers of balance efficiency. Three factors causing the deterioration of balance quality were analyzed: closing eyes, bending the head back and patient age. The influence of the given factors was measured using the significance *p* of the t-Student test and Cohen's *d* effect size and applied to differences for the logarithms of three of the mentioned above variables measured without and with the given deteriorating factor. In the majority of comparisons, the proposed new parameters of balance quality possessed higher statistical power to detect deteriorated balance quality than the standard parameters: standard deviation of the signal and ellipse area covering 90% of the signal envelope. Most valuable are the velocity and acceleration of CoPM for the medio-lateral direction. Logarithms of the analyzed parameters are proposed to be used in analyses because they possess normal or close to normal distribution and they are less sensitive to single high values occurring often in measurements.

## Introduction

The main problem at the current level of the development of neuroscience is to find diagnostic criteria for various disorders of the human balance system. Posturography is a very simple study that can be carried out without any major problems in any neurological clinic. A digital analysis of the posturographic signal creates a number of possibilities for extracting various parameters of this signal, which may have diagnostic values for various disorders. However, previous research on this signal has not yet yielded any valuable results. In order for a parameter to have a diagnostic value, it must discriminate against a healthy group from a group of ill patients. The large spread of the basic parameters (e.g. path length, mean velocity, ellipse area) analyzed in healthy and diseased groups and the large overlap of the ranges of parameter values in studied groups makes it impossible to use them as discriminators of disorders, despite the statistical significance of the parameters to differ between analyzed groups. Therefore, it is necessary to look for the causes of a large spread of posturographic parameters in healthy populations and to search for new parameters that would have lower spread and to discriminate against examined groups of patients better.

The current work presents the results of extracting new parameters from the posturographic signal and indicates their greater, though still not satisfying, diagnostic usefulness.

### Postural control system

The postural control system is a complex neuromuscular structure which regulates human body balance while quiet standing or performing different simple or complex tasks [1–4]. The nature of this regulation is broadly analyzed; however, the details of this regulation are still unclear.

The system is regulated by 4 main input information sources: (a) visual system [5–10] (b) vestibular system [11–14], (c) proprioceptive system [13, 15, 16] and alternatively by (d) conscious attention [17]. These inputs meet together in the subcortical and cortical structures of the Central Nervous System. The main part of this system is the cerebellum which performs the majority of input signal analysis. As an output of the system, corrective nerve impulses are generated, which regulate the basal tension and smooth regulatory contractions of the individual muscles of the human body.

Quiet standing for people is basically a simple task for the human balance system, thus, this case is often analyzed as the most elementary state of the regulation process. However, even this state is a very complex task [18].

The main regulatory problem consists in keeping the Center of Mass (CoM, called also Center of Gravity) over the center of the feet rectangle. This is performed mainly by modulating the Center of Pressure (CoP, posturographic signal) of the body by changing the tension of different ankle muscles [19]. A good understanding of this process is necessary in order to find effective methods for the diagnosis and treatment of postural control disturbances or supporting ill people with their balance problems.

### Posturographic signal

The posturographic signal is a 2-dimensional signal representing body sway during quiet standing or when performing different body or mental tasks. It represents the path of the ‘Center of Pressure’ over the ground. The registration can be performed both with eyes open (Eo) or eyes closed (Ec) to turn off the visual input, with the head in a normal position (Hn) or bent back (Hbb) to partially reduce the influence of the vestibular input, and while standing on hard ground or on a foam to reduce the influence of the proprioceptive input. The reduction of the influence of subsequent input systems deteriorates the general precision of body balance which is visible as an increase in values of the measured parameters describing the path oscillation size such as e.g. standard deviation of the signal or ellipse area covering the 90% of the signal envelope.

Similar to other organs, the human postural control system undergoes the ageing process [20, 21]. Young persons are able to perform balance with relative good efficiency even when excluding visual and vestibular input and while performing mental tasks which reduce the influence of conscious attention. On the other hand, the efficiency of the balance of old persons often deteriorates even if all input systems are active and the conscious attention supports the process of balance. Thus, the measurement of the precision of the balance control system requires the influence of subsequent input systems to be analyzed and balance to be performed when these systems are turned off or reduced (eyes closed/head bent back/ standing on a foam/mental tasks).

Body balance takes place in two axes: antero-posterior (AP) and lateral (LAT). Balance control is to a large extent independent between these axes. The independence ratio measured using a Principal Component Analysis is very high particularly in young persons and slightly lower in old persons with idiopathic gait disturbances [21].

### Stages of body balance regulation

In the case of AP direction, at least 3 stages of body balance control can be proposed. The common relations between them have been, however, poorly analyzed, until now.

Stage 1: Modulation of Achilles tendon tension (m. gastrocmemius, m. soleus)–balance is performed over the polygon defined by the heels and heads of metatarsal bones I and V. The increase in Achilles tension causes the shift of CoP forwards, in front of CoM, causing the gravitational force to act on CoM backwards. It is the most fundamental regulation process which dominates in healthy men [22].

Stage 2: a) An increase in the tension of flexors digitorum which together with the increase in Achilles tension increases the ground area of balance into the polygon: heels–toe tips. After flexors’ contraction the toes move down and the CoP shifts towards the toes. This kind of balance takes place if the forward sway of the CoM comes too close to the heads of the metatarsal bones. The forward shift of the CoP makes it possible to initiate the quicker movement of the CoM backwards.

b) The increase in the tension of frontal ankle muscles (m. tibialis anterior, m. extensor digitorum longus) which move the metatarsus up and shift the CoP toward heels. This kind of balance takes place if the CoM backward sway shifts towards the heels and the CoP must be shifted maximally back to initiate the forward movement of the CoM.

Stage 3: The opposite shift of hips and chest [23]. This 2-mass movement makes it possible to perform a quick shift of the CoM independently of the CoP position. The hips move back and the chest moves forward. The sum of the torques shifting these 2 masses is 0. However, the lower mass (hips) performs the longer shift backwards than the chest forwards. It results in a quick back shift of CoM which prevents falling forwards. The opposite action is performed if the CoM shifts too close to the heels.

In the case of the medio-lateral regulation, similar stages can be defined:

Stage 1: Modulation of the tibialis anterior and sagittal muscles. The tibialis anterior moves the medial metatarsus up and shifts the CoP of a given foot in a lateral direction. Sagittal muscles move the lateral part of the metatarsus up and shift the CoP in a medial direction. This regulation takes place without significant change of the total load between the legs.

Stage 2: The change of the tension of hip muscles which move the left/right hip up. This regulation changes the load between the legs and performs the longer shift of the CoP in the desired direction.

Stage 3: 2-mass regulation. The hips and chest move aside in opposite directions (e.g. hips to the left and chest to the right). This movement quickly shifts the CoM in the direction of the hip movement (left). The idea of the CoM correction is similar to that in the AP direction. This kind of regulation takes place especially if the CoM draws on the side border of the feet rectangle and the CoP shift may be insufficient to prevent falling down aside.

### The problem of CoM extraction

The CoM path estimation is not a trivial problem. Three main approaches to this problem were presented and discussed by Lafond [24]: the kinematic method [25, 26], the zero-point-to-zero-point double integration technique [27, 28] and the COP low-pass filter method [29].

The first one consists in simultaneous measurement of the movement of all body segments to estimate the instant CoM locations. The second one takes use of the horizontal ground force *F*_*hor*_ and assumes no acceleration of CoM in *F*_*hor*_ = 0 instants. In this method, the CoM and CoPM components were called: rambling and trembling, respectively.

The third method for CoM extraction for 1-mass model was presented by Caron [29] and further developed by Duarte at al. [30]. It does not need any additional measured parameters instead of the mass and height of the person under study.

In this model, the balance during quiet standing can be analyzed by using the inverted pendulum model in which the body is modeled by a stiff rod performing movements only in the ankle joints. In this model, the instantaneous acceleration of the CoM sway is proportional to the difference between the CoP and CoM: (CoPM = CoP—CoM) [3, 19, 31, 32] which is also proportional to the horizontal component of the gravitational force *F*_*g*_ = *mg · x*_*CoPM*_ /*h*_*e*_ (*h*_*e*_—height of the center of mass over the ground). Muscle contractions regulating body balance generate the shifts of the CoP and in this way modulate the acceleration of CoM.

If the CoP and CoM paths cross to each other, the body is in relative equilibrium because the gravitational force acting on CoM is equal to 0. These crossing points are called here Zero-Crossing (ZC) points. These points, however estimated using another method, were also called the Instant Equilibrium Points (IEP) [27, 28, 30].

In this method, the CoM is extracted from the CoP using a digital filter described by the equation:

$$\begin{array}{l} \frac{CoM(j\omega )}{CoP(j\omega )}=\frac{\omega_{0}^{2}}{\omega^{2}+\omega_{0}^{2}}=\frac{g/h_{e}}{\omega^{2}+g/h_{e}}\\ \omega_{0}=\sqrt{\frac{mgh_{e}}{I}},\;h_{e}=h\cdot \xi ,\;\xi =1.15 \end{array}$$ (1)

*ω*_*0*_ is the natural frequency of the pendulum which depends on the body mass *m* and on the moment of inertia *I*. ξ is the factor representing the individual shape of the body and is equal to about 1.15. The basis for deriving this formula was the assumption that the momentary acceleration of the resultant CoM path must be proportional to the CoPM.

It has been proved that the regulation of the balance is performed by sending impulses to muscles and the rate of impulses to Achilles tendon muscles has been estimated to be about 2.4–2.6 per second [22]. If *x*_*CoM*_ moves to some border of the feet rectangle, the muscle contraction impulse shifts the CoP outside the CoM point (relative to the center of feet) and the gravitational force starts first to slow down *x*_*CoM*_ movement, and next, to turn back its direction. If the corrective impulse (or the series of corrective impulses) is finishing, *x*_*CoP*_ goes back approximately towards the center of feet.

### Exemplary CoM/CoPM decomposition

**Fig 1:**
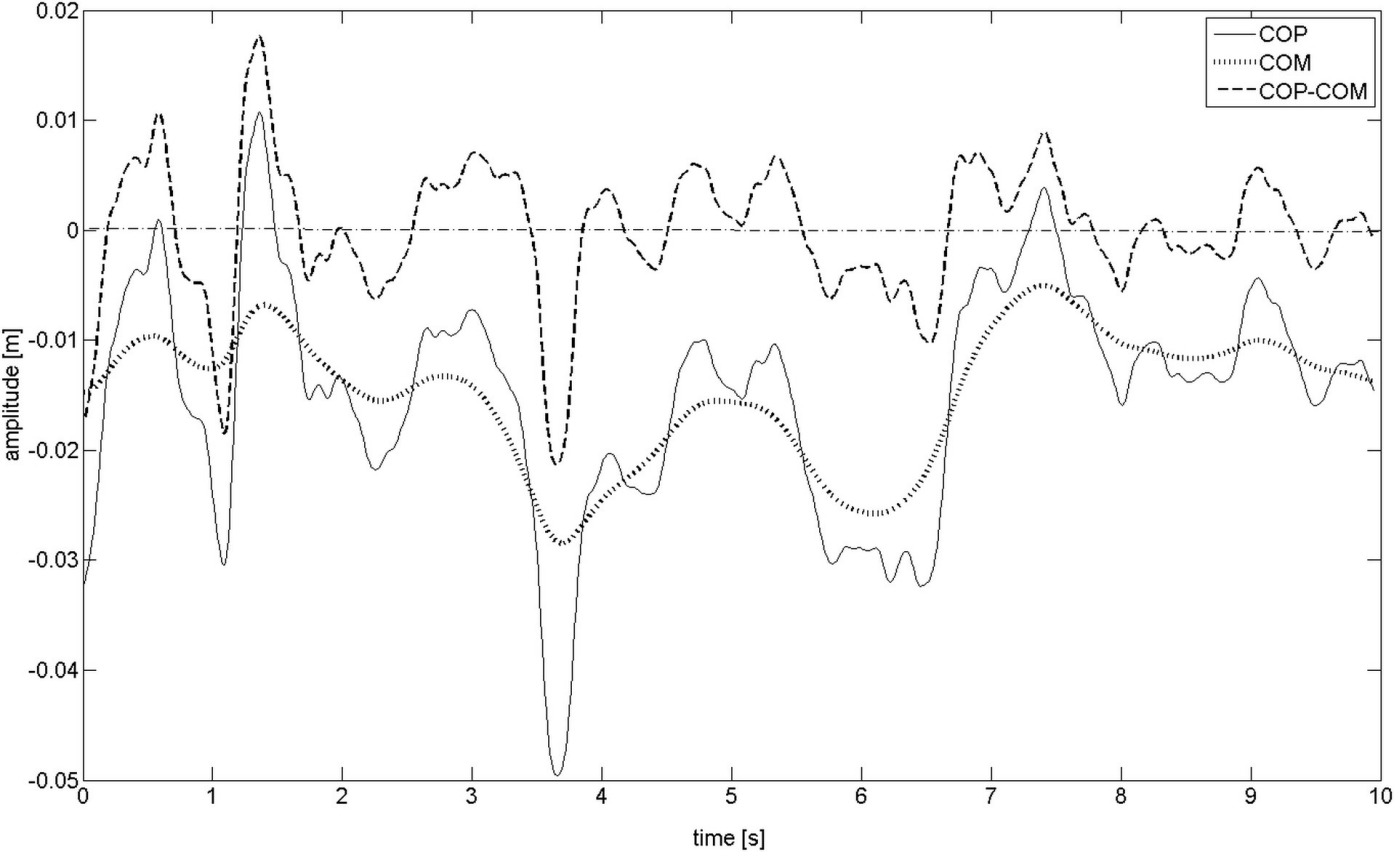
The exemplary decomposition of the posturographic signal (Center of Pressure, CoP) into Center of Mass (CoM) and difference between CoP and CoM (CoPM) signals.

"Fig 1" shows the exemplary decomposition of the CoP (posturographic) signal into CoM and CoPM using Eq 1. Let us analyze this figure. The CoM path possesses the characteristics of slow drift. The number of maxima during a 10s trial is 5–6 which corresponds to the main frequency of about 0.4–0.5 Hz. The CoPM signal represents the oscillations of CoP surround CoM. Digital analysis shows that the positive value of the estimated CoPM is precisely proportional to the negative acceleration of the CoM. The number of maxima in the exemplary 10s-trial of *x*_*CoPM*_ signal is about 30–40 which corresponds to the main frequency of about 3–4 Hz. It can be carefully concluded that about 3–4 muscle impulses per second modulate human body balance which stands in general agreement with Loram's et al. [22, 33] measurements of 2–3 impulses per second of gastrocnemius and soleus muscles registered using the adopted USG method. It must be pointed out that different impulses may come from different muscles. Next, it can be observed that the number of zero crossings in the CoPM signal is about 20. This means that the mean duration of the elementary front and back correction of CoM lasts about 1s and about 1–2 impulses control the elementary front/rear correction. It can be concluded that the higher number of ZCs denotes the corrective impulses to the muscles to be more often stronger than the optimum ones which causes statistically a quicker change of correction direction (incidence of ZC).

The optimum muscle contraction impulse can be defined to be a contraction that stops *x*_*CoM*_ and *x*_*CoP*_ in the equilibrium point. In this point, *x*_*CoM*_ = const, *v*_*CoM*_ *=* 0, *a*_*CoM*_ *= 0* and by consequence *x*_*CoPM*_ *= 0*. This theoretical, unstable point is proposed to be termed the 'superstability point' and it corresponds to a pencil standing on a spike.

The superstability point reflects the ideal and optimal body balance regulation state, thus, the paramters of ZC points seem to be the interesting and encouraging parameters to possess the discrimination power between healthy and ill persons.

A close to superstability point can be observed in "Fig 1" for the time *t* = 8-9s. The impulses being too weak are not able to change the direction of the *x*_*CoM*_ movement [1]. After such a corrective impulse finishes, *x*_*CoM*_ follows the falling down movement in the same direction which requires the next corrective impulse trying to stop this *x*_*CoM*_ falling. These impulses do not generate the ZC incident (see "Fig 1" exemplary fragments *t* = 2.5–3.4 s and *t* = 5.5–6.5 s. On the other hand, too strong an impulse (or a series of impulses) causes *x*_*CoM*_ to move in the opposite direction. It requires, the next, *x*_*CoP*_ to be shifted to the opposite side of *x*_*CoM*_ to stop a falling down movement in the opposite direction.

One of the main problems connected with the posturographic signal analysis is the determination of the valuable parameters which would effectively determine the real quality of balance control and which would be independent of the tiredness of the patient and/or the stiffness of the body during balance (e.g. stand to attention vs. relaxed standing).

The majority of research being presented in the area of the analysis of posturographic signals shows results for the CoP path only which is significantly charged with the slow drift of CoM. Thus, results, however, presenting statistical significances between the different groups analyzed, do not present diagnostic values, due to the large spread of the data that surround their mean values. The decomposition of the CoP path into CoM and CoPM reduces the influence of the slow CoM drift in the resultant CoPM path, thus, it is expected that CoPM oscillations and especially ZC parameters should better distinguish between the different groups analyzed than the CoP.

### The values of *v*_*CoM*_, *v*_*CoPM*_ and *a*_*CoPM*_ in the ZC points

The perfect impulses generating superstability separate the impulses to those being too weak and being too strong [1]. Thus, the velocity and acceleration in ZC points could be the estimators of the precision of body balance regulation. Higher mean values are expected to be markers of deteriorated body balance and close to zero values are proposed to be markers of the occurrence of perfect corrective impulses.

The aim of the study was to estimate the value of the parameters connected with the ZC points as markers of postural control precision.

### Hypothesis

The new proposed estimators of balance quality are the velocity of CoM (*v*_*CoM*_) and velocity and acceleration of CoPM (*v*_*CoPM*,_ *a*_*CoPM*_) in the ZC points.

The velocities of CoM and of CoPM are represented by tangents of the *x*_*CoM*_ and *x*_*CoPM*_ curves in ZC points. The values of *v*_*CoPM*_ should be as small as possible in order not to go too quick to the opposite side of regulation. Higher *v*_*CoPM*_ requires stronger braking on the opposite side which requires stronger corrective muscle impulses performing it. The higher value of *v*_*CoM*_ in the ZC point denotes the braking of *x*_*CoM*_ to be turned on later. Acceleration of CoPM in the ZC point represents mainly the force connected with the actual corrective impulse, because the gravitational force is equal to 0 in ZC points. In the ideal case, the *a*_*CoPM*_ should be equal to 0 because the force generated by the corrective impulses should finish exactly in the ZC point and start to act in the opposite direction after crossing the ZC point. The higher variability of *a*_*CoPM*_ values surround 0 are expected to be connected with less accurate balance regulation. *a*_*CoM*_ is equal to 0 in the ZC point due to the elementary assumption of the Eq (1): *a*_*CoM*_ ~ *x*_*CoPM*_. Thus, small, close to 0 values of v_*CoM*_, *v*_*CoPM*_ and *a*_*CoPM*_ are expected in the optimal balance control. Very small values of all 3 parameters represent the moment of reaching the close to superstability regulation.

## Materials and methods

### Subjects

In order to analyze the significance of *v*_*CoM*_ / *v*_*CoPM*_ / *a*_*CoPM*_ parameters in the ZC points to be the estimators of balance quality, their values have been calculated for 384 persons at different ages, healthy or possessing unclassified gait disorders. The posturographic signals were registered in 4 Eyes/Head conditions: Eyes Open/Closed (Eo vs.Ec) vs. Head Normal/Bent Back (Hn vs. Hbb). Posturographic signals were recorded in the Neurological Department of the Medical University in Luebeck. The investigation was performed according to the principles of the Declaration of Helsinki and was approved by Ethics Committee at the Medical University in Luebeck. The written consent was obtained from subjects after the aim of the procedure was explained and all the data were analyzed anonymously. The subjects with gait disturbance were recruited from the patients of the Neurological Department of the Medical University in Luebeck in 1998–2000. The 50–79 years old persons were recruited from the cohort of the EPOS program (European Prospective Osteoporosis Study) which was conducted since 1989 in 18 European countries. Young persons (group H3) were recruited from the students and workers of the Medical University in Luebeck. The majority of persons belonged to the population of the city of Luebeck. Only persons who were able to perform all 4 registration conditions (Eo/Ec/Hn/Hbb) were included to the analysis. Data were earlier analyzed by Stolze et al. [34]. The patients were asked to stand quietly on a posturographic platform. In the case of the Eo condition, they were asked to look at a fixation point that was placed at a distance of 2 m. In the case of the Hbb condition, patients were asked to bend maximally the head back while registering the signal. The patients were divided into 4 groups: ‘Old with Gait Disturbance’ (group GD, *N* = 54, age 81,9±6,5), ‘Healthy Old’ (group H1, *N* = 98, age 76,5±4,1), ‘Healthy Middle Aged’ (group H2, *N = 193*, age 61,6,5±5,2) and ‘Healthy Young’ (group H3, *N* = 39, age 30,1±5,6). The patients in group GD either (a) reported non-specific gait disturbances resulting in stumbling or falling in the period of the previous six months or (b) were neurologically diagnosed as having an unexplained and unclassified gait disorder. Patients were rejected if the gait disorder could be explained by one or more of the following reasons: paraparesis, hemiparesis, tetraspasticity or tetraparesis, any kind of myelopathic, cerebellar, myopathic, vestibular, brainstem or neuropathic lesions, any degenerative disease of the peripheral or central motor system, intake of CNS-relevant drugs and any medical, dermatologic, or orthopedic dysfunction interfering with gait. Normal, healthy patients who did not exhibit any deviations in the neurological examination joined groups H1–H3. The healthy persons had to present an inconspicuous gait pattern and had to be able to perform six tandem steps without deviation during at least one out of two subsequent trials.

### Apparatus

The posturographic platform from the Toennies Company was used. Two feet markers at a 6cm distance were marked on the platform surface. Four force sensors placed in corners of the plate registered the force signals and the signals were sent to the PC interface. Device calibration was performed before each measurement.

### Procedure

The analyzed posturographic signals were 20.48 s long and they were sampled with a frequency of 50 Hz. The signals were preliminary filtered using a low pass 4th order Butterworth filter with filter frequency *f* = 10 Hz. After the filtering procedure, the *x*_*CoP*_ signals were decomposed into *x*_*CoM*_ and *x*_*CoPM*_ using Formula 1. Next, the ZC instants were determined and the values *v*_*CoM*_^*ZC*^, *v*_*CoPM*_^*ZC*^ and *a*_*CoPM*_^*ZC*^ were estimated for every ZC point. The mean values *V*_*CoM*_ and *LV*_*CoPM*_ over the given signal were calculated in a normal and logarithmic scale: *V*_*CoM*_ = mean(|*v*_*CoM*_^*ZC*^|) and *LV*_*CoM*_ = mean (log_10_ (|*v*_*CoM*_^*ZC*^| ) ). In the case of CoPM acceleration, the parameters *A*_*CoPM*_ = mean (|*a*_*CoPM*_^*ZC*^| ) and *LA*_*CoPM*_ = mean (log_10_ (|*a*_*CoPM*_^*ZC*^| ) ) were estimated.

In order to determine if the new parameters are better estimators of balance quality, the standard *x*_*CoP*_ and *x*_*CoPM*_ oscillations were estimated using the formulas: *S*_*CoP*_ = std(*x*_*CoP*_), *S*_*CoPM*_ = std(*x*_*CoPM*_) and in the logarithmic scale: *LS*_*CoP*_ = log_10_ (std(*x*_*CoP*_) ), *LS*_*CoPM*_ = log_10_ (std(*x*_*CoPM*_) ). The second standard parameter was the ellipse area *EA*^*90*^ covering 90% of the 2D path envelope and the logarithm of this area *LE =* log_10_(*EA*^*90*^). The logarithms of the estimated parameters have been used, because, as presented later in the Results section, the distribution of all the parameters is log-normal rather than normal. Additionally, two simple functions of *v*_*CoM*_^*ZC*^, *v*_*CoPM*_^*ZC*^ and *a*_*CoPM*_^*ZC*^ were analyzed: *LM* = log_10_ (|mean(*v*_*CoPM*_^*ZC*^ ·*a*_*CoPM*_^*ZC*^ · *v*_*CoPM*_^*ZC*^)| ) and *ML* = mean (log_10_(|*v*_*CoM*_^*ZC*^*· v*_*CoPM*_^*ZC*^ · *a*_*CoPM*_^*ZC*^| ) ).

Three factors deteriorating balance quality have been analyzed: closing eyes (eyes factor, EF), bending the head back (head factor, HF) and patients age (age factor, AF). The statistical significances *p* were estimated for *LS*, *LE* and *LV*_*CoM*_, *LV*_*CoPM*_ and *LA*_*CoPM*_ parameters using t-Student test. The paired samples t-Student test (Matlab’s *ttest* function) was used for the analysis of EF and HF factors and the independent samples t-Student test (Matlab’s *ttest2* function) was used for the AF factor, respectively. The default TAIL = ‘both’ parameter was used in both functions. The Cohen's *d* effect size was calculated using formula: *d* = |*u*_*1*_*-u*_*2*_|/*s*, *s*^*2*^ = ((*n*_*1*_-1)*s*_*1*_+(*n*_*2*_-1)*s*_*2*_ ) / (*n*_*1*_*+n*_*2*_-2), where *u*_*i*_, *s*_*i*_ and *n*_*i*_ stand for the mean, standard deviation and number of elements of the data sets compared. The analysis was performed separately for AP and LAT signals. The analysis for *V*_*CoM*_ and *V*_*CoPM*_ is not presented because it showed a lower significance than *LV*_*CoM*_ and *LV*_*CoPM*_ and it is formally incorrect due to the strongly non-Gaussian distribution.

## Results

### Distribution of the analyzed parameters

**Fig 2:**
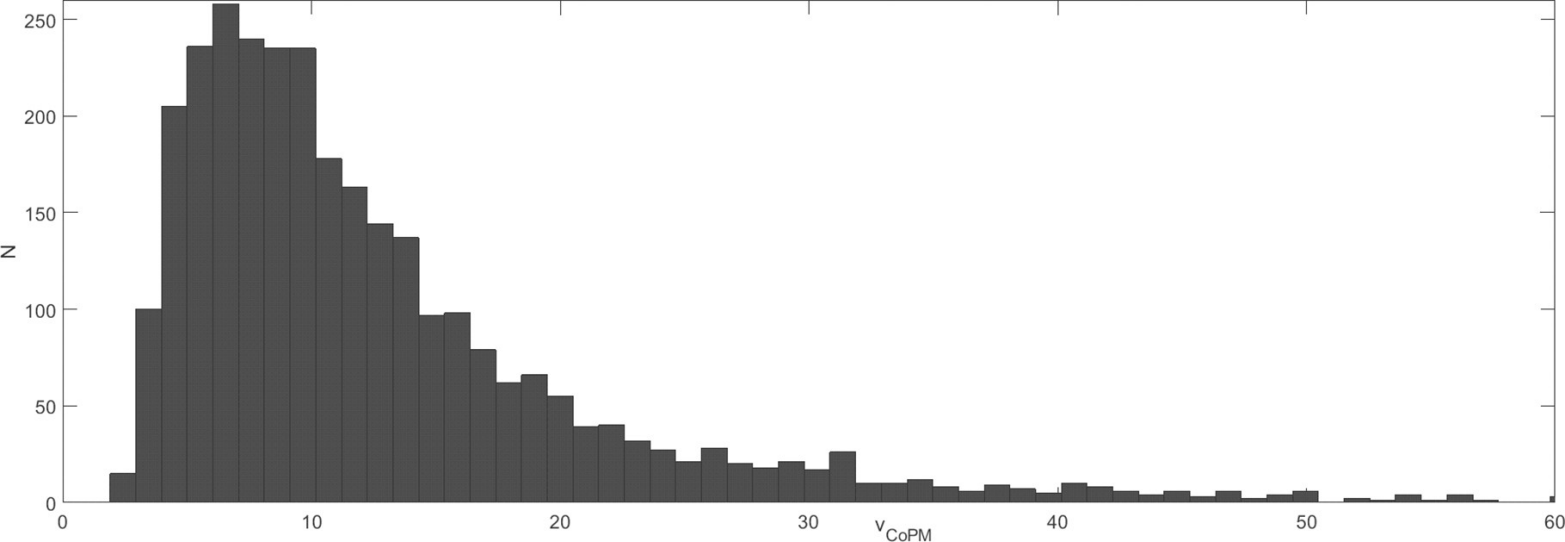
The exemplary distribution of the variable *V*_*CoPM*_ = mean(*v*_*CoPM*_^ZC^). *v*_*CoPM*_^ZC^ denotes the value of *v*_*CoPM*_ in the given ZC point and *V*_*CoPM*_ denotes the mean over all the ZC points in the given signal. Values for all patients and their registration conditions are presented. The distribution is log-normal rather than normal.

**Fig 3:**
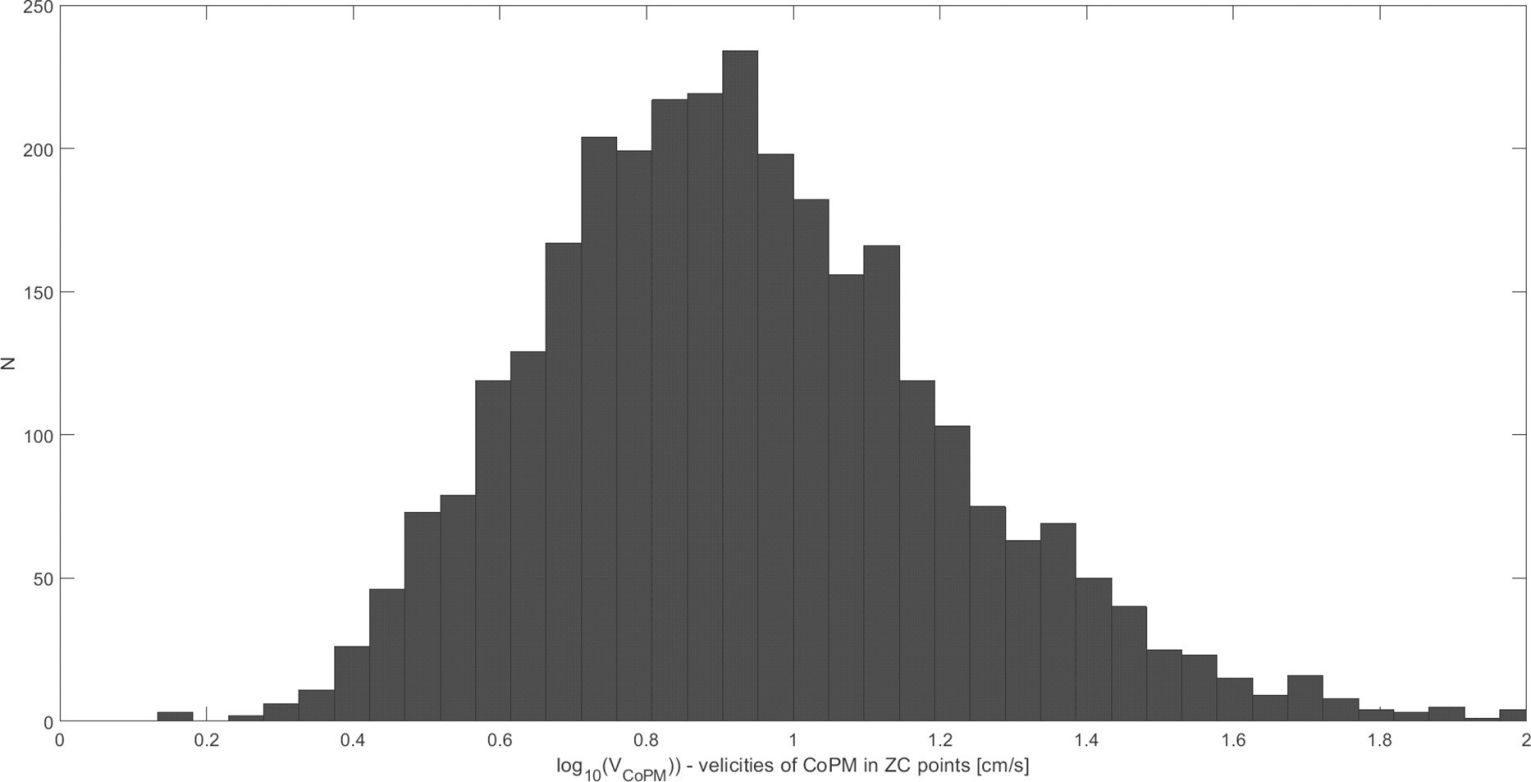
The exemplary close to normal distribution of the variable *LV*_*CoPM*_ = mean(log_10_ *v*_*CoPM*_^ZC^). *v*_*CoPM*_^ZC^ denotes the value of *v*_*CoPM*_ in the given ZC point and *LV*_*CoPM*_ denotes the mean over all the ZC points in the given signal. Values for all patients and their registration conditions are presented.

**Fig 4:**
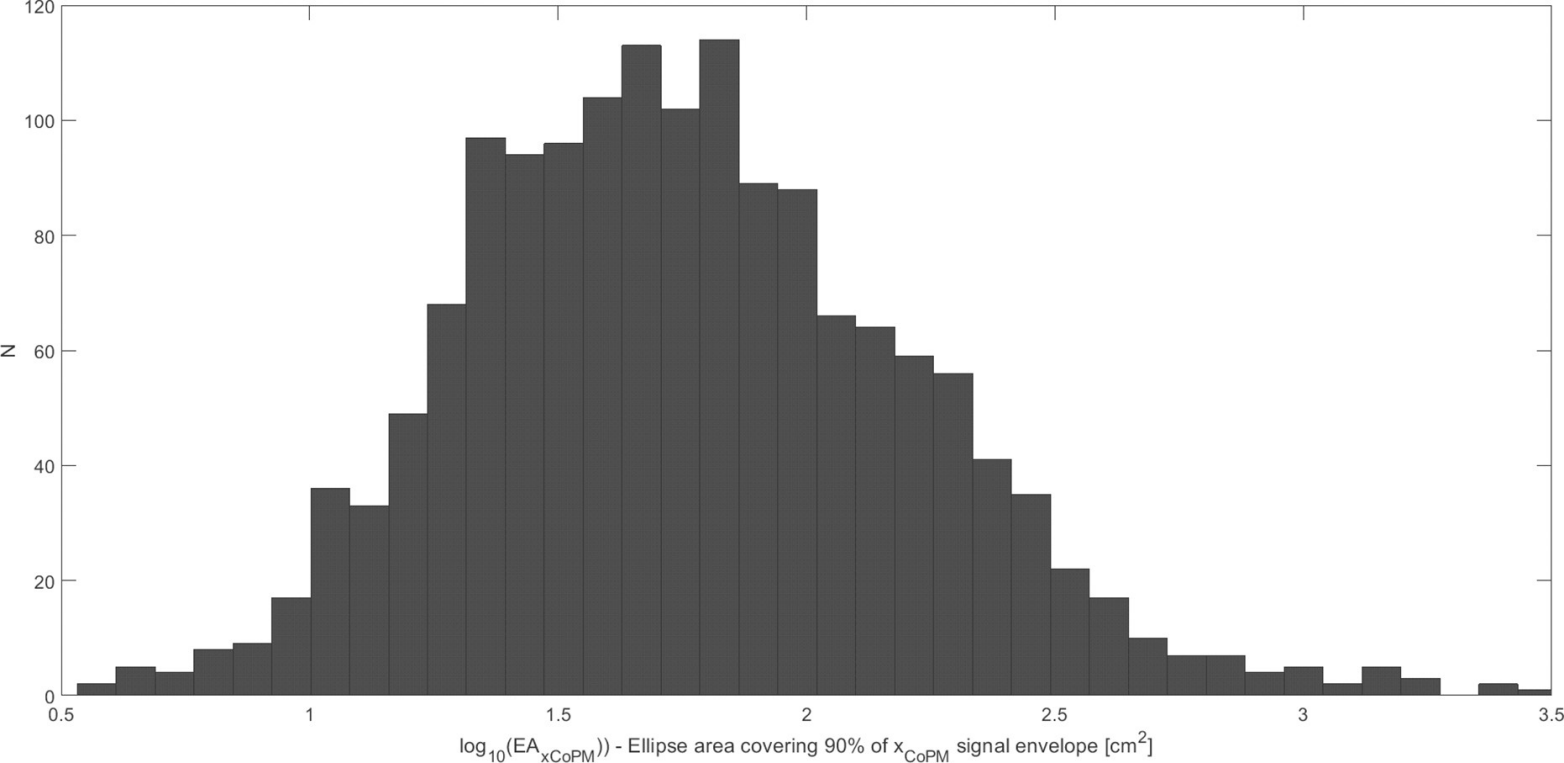
The exemplary close to normal distribution of the logarithm of Ellipse Area covering 90% of the 2D signal envelope (*LE*). Values for all patients and their registration conditions are presented.

In the first step, the estimated parameters were analyzed using the normality Shapiro-Wilk test to verify their distribution to be normal or not normal. The normality of the distribution has been rejected for all analyzed parameters in subsequent age groups and registration conditions. The visual inspection into the distribution of the individual parameters shows their tendency to be rather log-normal than normal (see the exemplary distribution of *V*_*CoPM*_ in "Fig 2"). Thus, the analysis has been repeated for the logarithms of the individual parameters analyzed. The exemplary distributions for the logarithms of the parameters are presented in "Figs 3 and 4". "Fig 3" shows the close to normal distribution of *LV*_*CoPM*_, and "Fig 4"–for *LE*_*CoP*_. Similar tendencies have been observed for all of the analyzed parameters.

The Shapiro-Wilk normality test, when preformed in individual patient groups and registration conditions, shows the distribution of the analyzed logarithmic parameters to be normal in the majority of the groups analyzed. Thus, the logarithms of individual parameters are recommended in future analyzes as being less sensitive to the individual high values of the measured parameters. The statistical significances of parameters' differences for subsequent balance deterioration factors are presented in "Table 1". The values of the Cohen's *d* effect size are presented in "Table 2".

**Table 1:** The statistical significances for differences between pairs of signals representing the given deteriorations of postural control quality. ZC–Zero-Crossing points, CoM–Center of Mass signal, CoPM–difference between Center of Pressure and Center of Mass signals, AP–antero-posterior direction, LAT–lateral direction, EF–closing eyes deterioration factor, HF–bending head deterioration factor, AF–age/gait deterioration factor. The logarithms of *p* (log_10_ *p*, t-Student test) are presented (e.g. log_10_ 0.05 = -1.3, log_10_ 0.001 = -3). Eo–eyes open, Ec–eyes closed, Hn–head normal, Hbb–head bent back, GD–old with gait disturbance group, H1 –old healthy group, H2 –middle aged, H3 –young. *LS* = log_10_(std(*signal*)), *LE*—log_10_(*E*_*90*_), *E*_*90*_ –area of the ellipse covering 90% of the 2D signal envelope, *LV*_*CoPM*_ **=** mean (log_10_ (|*v*_*CoPM*_^*ZC*^| )), *LV*_*CoM*_ **=** mean(log_10_(|*v*_*CoM*_^*ZC*^|)), *LA*_*CoPM*_ **=** mean(log_10_(|*a*_*CoPM*_^*ZC*^|)), *v*_CoPM_^ZC^, *v*_CoM_^ZC^, *a*_*CoPM*_^*ZC*^–velocities and acceleration of CoPM and CoM signals in the ZC points. *LM* = log_10_ (|mean (*v*_CoPM_^ZC^ · *v*_CoM_^ZC^ · *a*_CoPM_^ZC^)| ), *ML* = mean (log_10_ (|*v*_CoPM_^ZC^ · *v*_CoM_^ZC^ · *a*_CoPM_^ZC^|) ). The logarithms of the individual parameters are used and recommended for use as possessing a normal or nearly normal distribution, thus, being less sensitive to single strongly high values. Velocity and acceleration of CoPM in the ZC points for lateral direction possess a better ability to detect the postural control deteriorations than the standard parameters: standard deviation of the signal (*LS*) and area of the ellipse (*LE*) covering 90% of the 2D signal envelope. The strongest significances in each column are marked in bold.

log_10_ *p*	Factors deteriorating balance quality						
	Eyes factor (EF)		Head factor (HF)		Age factor (AF)		
	HN, Eo vs. EC	HBB, Eo vs. EC	EO, Hn vs. Hbb	EC, Hn vs. Hbb	GD vs. H1	H1 vs. H2	H2 vs. H3
Standard parameters–AP							
LS_CoP-AP_	-17.5	-31.4	-0.6	-5.9	-9.3	-4.0	-2.6
LS_CoPM-AP_	-33.7	-51.5	-0.6	-2.6	-5.2	-5.8	-3.1
Zero Crossing based parameters–AP							
LV_CoPM-AP_	-42.8	-62.3	-0.1	-3.6	-3.7	-5.8	-6.8
LV_CoM-AP_	-13.5	-34.0	-1.2	-2.6	-5.9	-3.6	-1.5
LA_CoPM-AP_	-30.1	-38.4	-2.3	-5.7	-6.1	-13.1	-5.4
LM_AP_	-17.0	-22.2	-0.6	-1.6	-3.7	-5.5	-1.1
ML_AP_	-37.5	-59.5	-0.0	-5.1	-6.1	-8.4	-5.2
Ellipse parameters							
LE_CoP_	-40.8	-35.8	-6.7	-8.0	-5.2	-6.0	-3.7
LE_CoPM_	-43.3	-31.8	-13.9	-10.9	-6.0	-11.6	-2.0
Standard parameters–LAT							
LS_xCoP-LAT_	-33.8	-32,4	-6.1	-8.5	-1.0	-4.2	-4.3
LSx_CoPM-LAT_	-77.8	-66,6	-29.9	-23.0	-5,6	-13.6	-3.9
Zero Crossing based parameters–LAT							
LV_CoPM-LAT_	-79.3	-75.7	**-51.4**	**-40.8**	-9.3	-16.3	**-8.7**
LV_CoM-LAT_	-68.1	-60.6	-10.0	-5.5	-4.5	-6.1	-2.7
LA_CoPM-LAT_	-60.0	-49.0	-42.0	-32.7	**-11.1**	**-26.5**	-5.8
LM_LAT_	-40.0	-29.7	-18.1	-9.6	-7.8	-11.2	-2.0
ML_LAT_	**-87.1**	**-80.0**	-44.7	-33.5	-9.9	-19.2	-6.8

**Table 2:** The Cohen's *d* effect sizes for differences between pairs of signals representing the given deteriorations of postural control quality. Abbreviation's description—see "Table 1". Velocity and acceleration of CoPM in the ZC points for lateral direction possess a better ability to detect the postural control deteriorations than the standard parameters: standard deviation of the signal (*LS*) and area of the ellipse (*LE*) covering 90% of the 2D signal envelope. The strongest significances in each column are marked in bold.

*d*_*cohen*_	Factors deteriorating balance quality (Cohen *d* statistics)						
	Eyes factor (EF)		Head factor (HF)		Age factor (AF)		
	HN, Eo vs. EC	HBB, Eo vs. EC	EO, Hn vs. Hbb	EC, Hn vs. Hbb	GD vs. H1	H1 vs. H2	H2 vs. H3
Standard parameters–AP							
LS_xCoP-AP_	0,41	0,50	0,41	0,18	0,53	0,24	0,26
LS_xCoPM-AP_	0,57	0,69	0,57	0,11	0,39	0,30	0,29
Zero Crossing based parameters–AP							
LV_CoPM-AP_	0,61	0,68	**0,61**	0,13	0,31	0,30	0,46
LV_CoM-AP_	0,39	0,58	0,39	0,13	0,41	0,23	0,19
LA_CoPM-AP_	0,49	0,52	0,49	0,16	0,42	0,47	0,41
LM_AP_	0,50	0,55	0,50	0,12	0,32	0,29	0,16
ML_AP_	0,58	0,66	0,56	0,15	0,42	0,37	0,40
Ellipse parameters							
LE_xCoP_	0,71	0,62	0,26	0,23	0,39	0,31	0,33
LE_xCoPM_	0,76	0,62	0,39	0,29	0,42	0,44	0,23
Standard parameters–LAT							
LS_CoP-LAT_	0,71	0,63	0,27	0,25	0,15	0,25	0,36
LS_CoPM-LAT_	1,05	0,82	0,54	0,35	0,40	0,48	0,34
Zero Crossing based parameters–LAT							
LV_CoPM-LAT_	0,89	0,72	**0,61**	**0,45**	0,53	0,53	**0,53**
LV_CoM-LAT_	**1,09**	**0,90**	0,33	0,20	0,35	0,31	0,27
LA_CoPM-LAT_	0,67	0,51	0,51	0,38	**0,59**	**0,69**	0,42
LM_LAT_	0,81	0,61	0,49	0,30	0,48	0,43	0,23
ML_LAT_	0,96	0,76	0,55	0,39	0,55	0,58	0,46

### The values of the parameters in the analyzed groups

**Fig 5:**
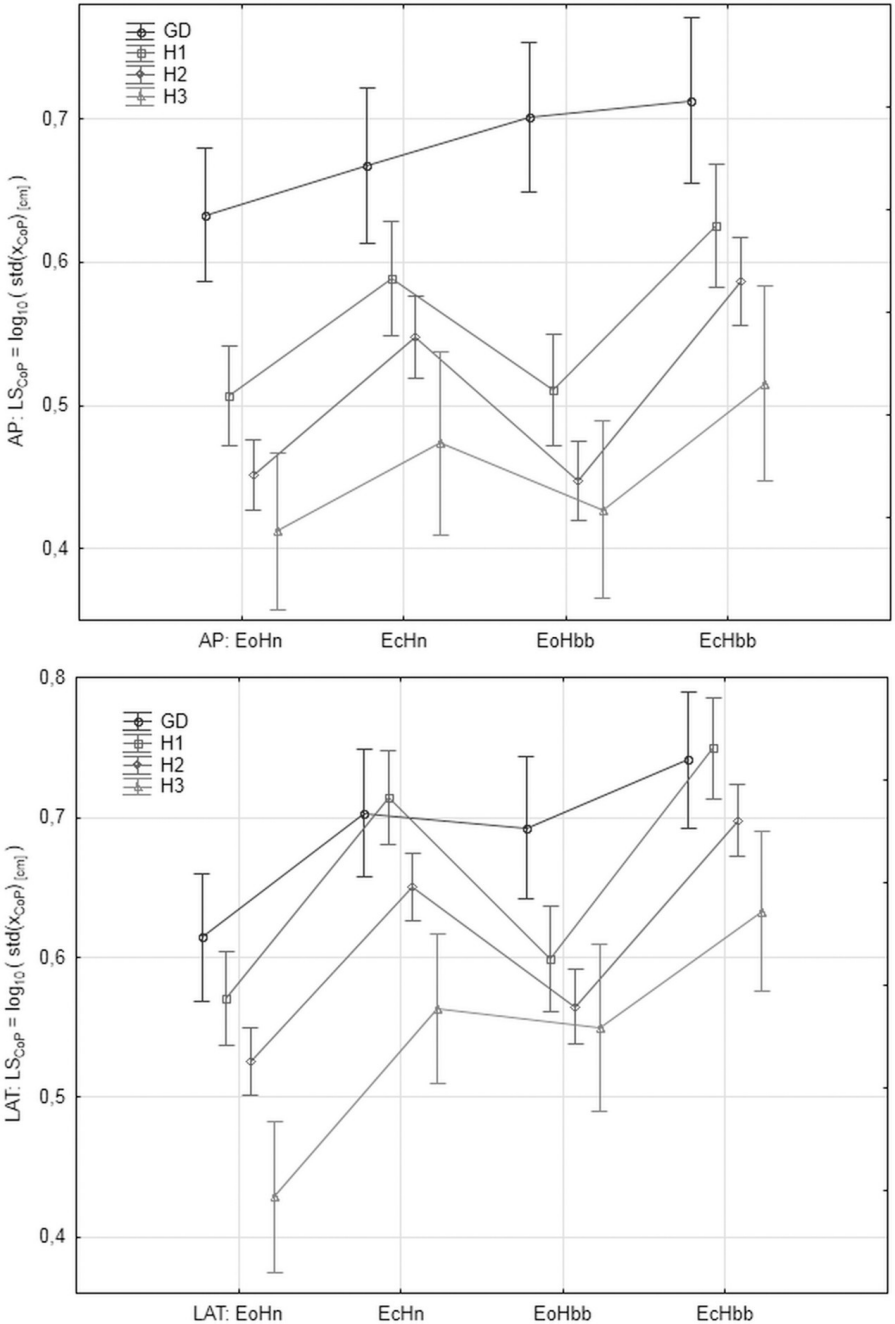
The mean values of *LS*_*CoP*_ = log_10_(*S*_*CoP*_) in the subsequent patients and registration condition groups. *S*_*CoP*_ represents the variability of *x*_*CoP*_ signal measured as *S*_*CoP*_ = std (*x*_*CoP*_). Upper: antero-posterior (AP), bottom: lateral (LAT) component of the signal.

**Fig 6:**
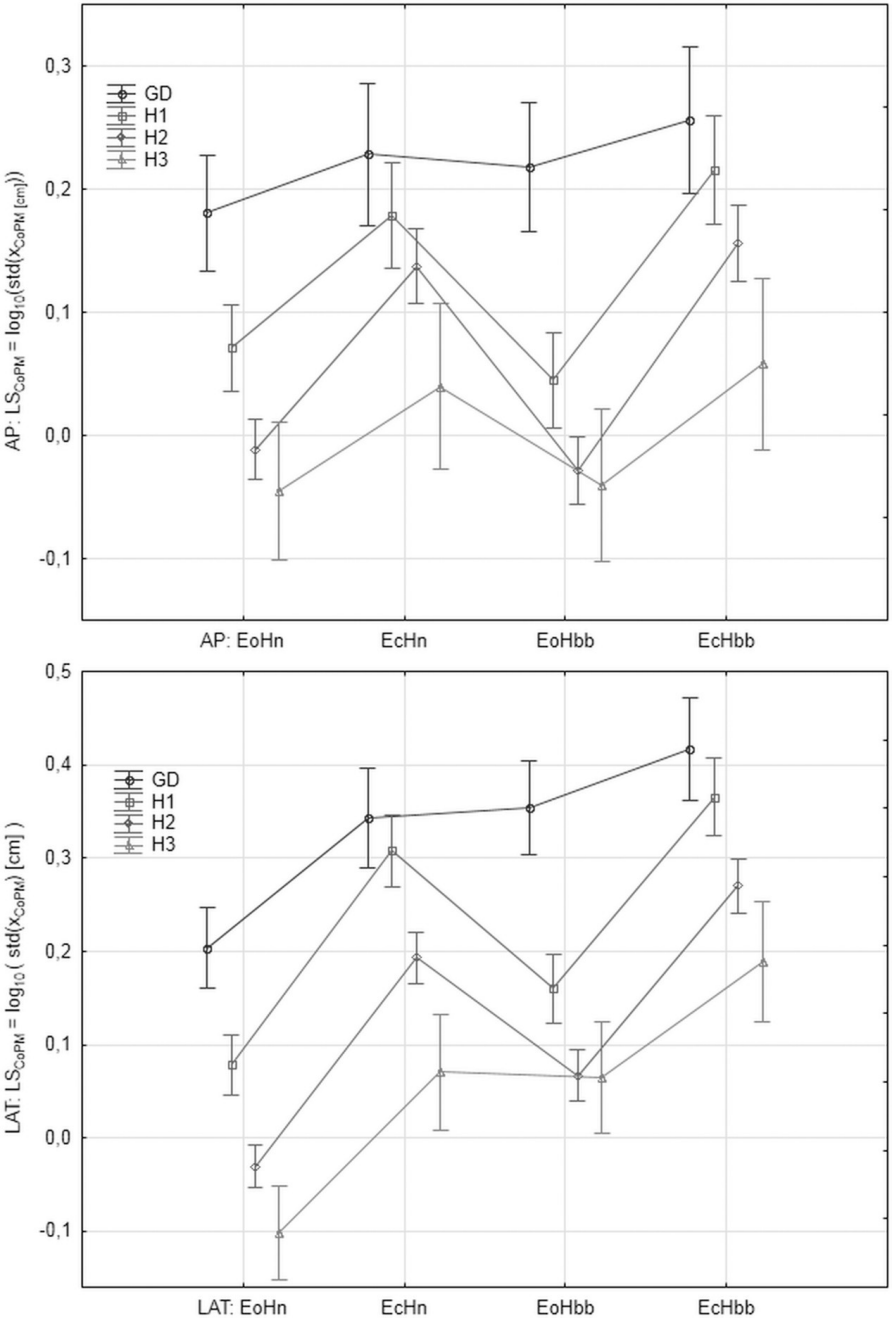
The mean values of *LS*_*CoPM*_ = log_10_(*S*_*CoPM*_) in the subsequent patients and registration condition groups. *S*_*CoPM*_ represents the variability of *x*_*CoPM*_ signal measured as *S*_*CoPM*_ = std (*x*_*CoPM*_). Upper: antero-posterior (AP), bottom: lateral (LAT) component of the signal.

**Fig 7:**
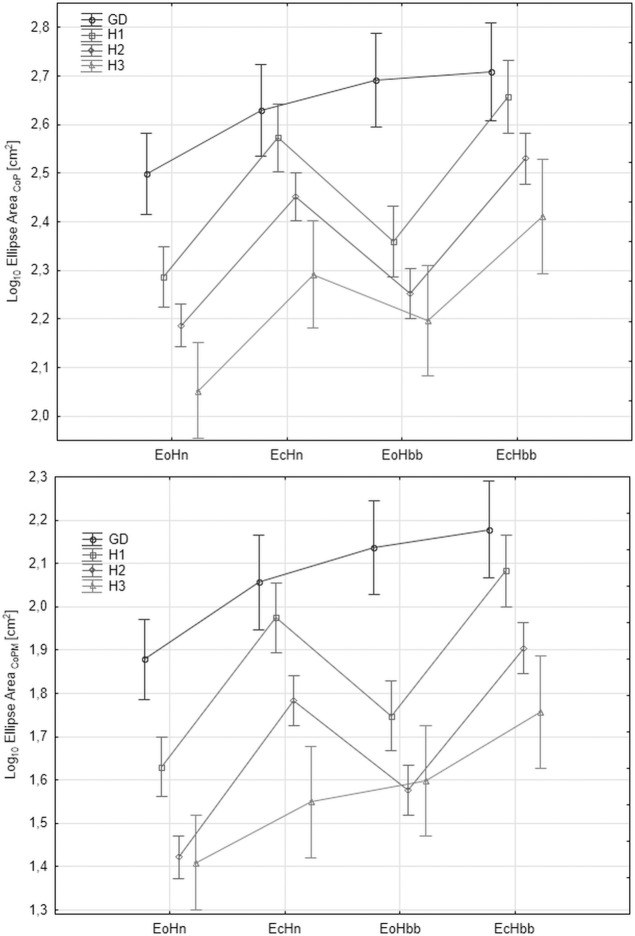
The mean values of *LE*_*CoP*_ = log_10_(*E_CoP_^90^*) (upper) and *LE*_*CoPM*_ = log_10_(*EA_CoPM_^90^*) (bottom) in the subsequent patients and registration condition groups.

"Fig 5" shows the values of *LS*_*CoP*_ in the analyzed age groups and registration conditions for AP and LAT signal components, respectively. "Fig 6" shows the mean values of *LS*_*CoPM*_, respectively. "Fig 7" shows the values of the *LE* parameter. The markers in graphs denote the standard error of the mean in the analyzed group.

**Fig 8:**
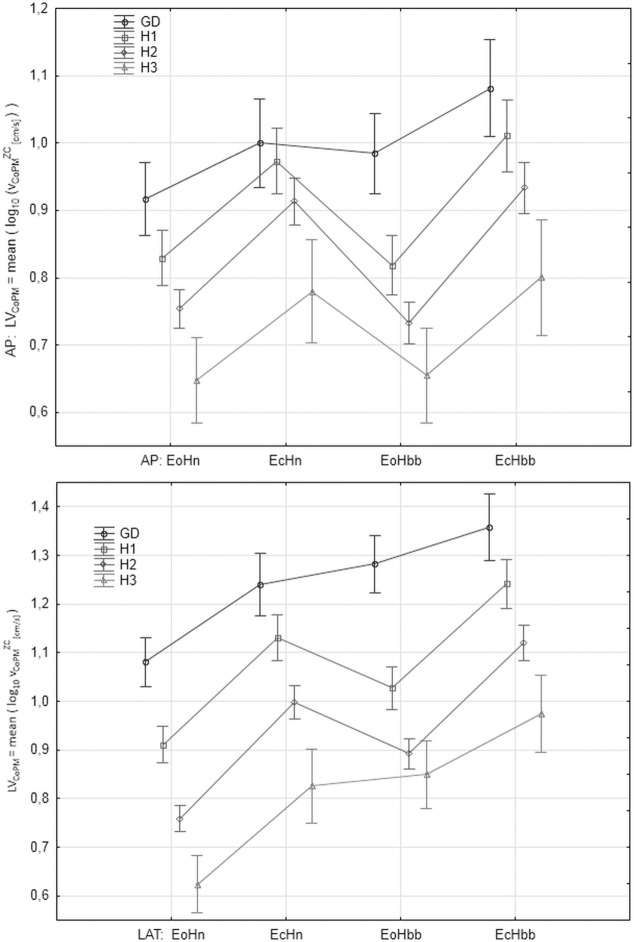
The mean values of *LV*_*CoPM*_ = mean(log_10_(*v_CoPM_^ZC^*)) in the subsequent patients and registration condition groups. *v*_*CoPM*_^*ZC*^ represents the velocity of *x*_*CoPM*_ signal in the given Zero-Crossing point. Upper: antero-posterior, bottom: lateral component of the signal. Markers denote the standard error of the mean.

**Fig 9:**
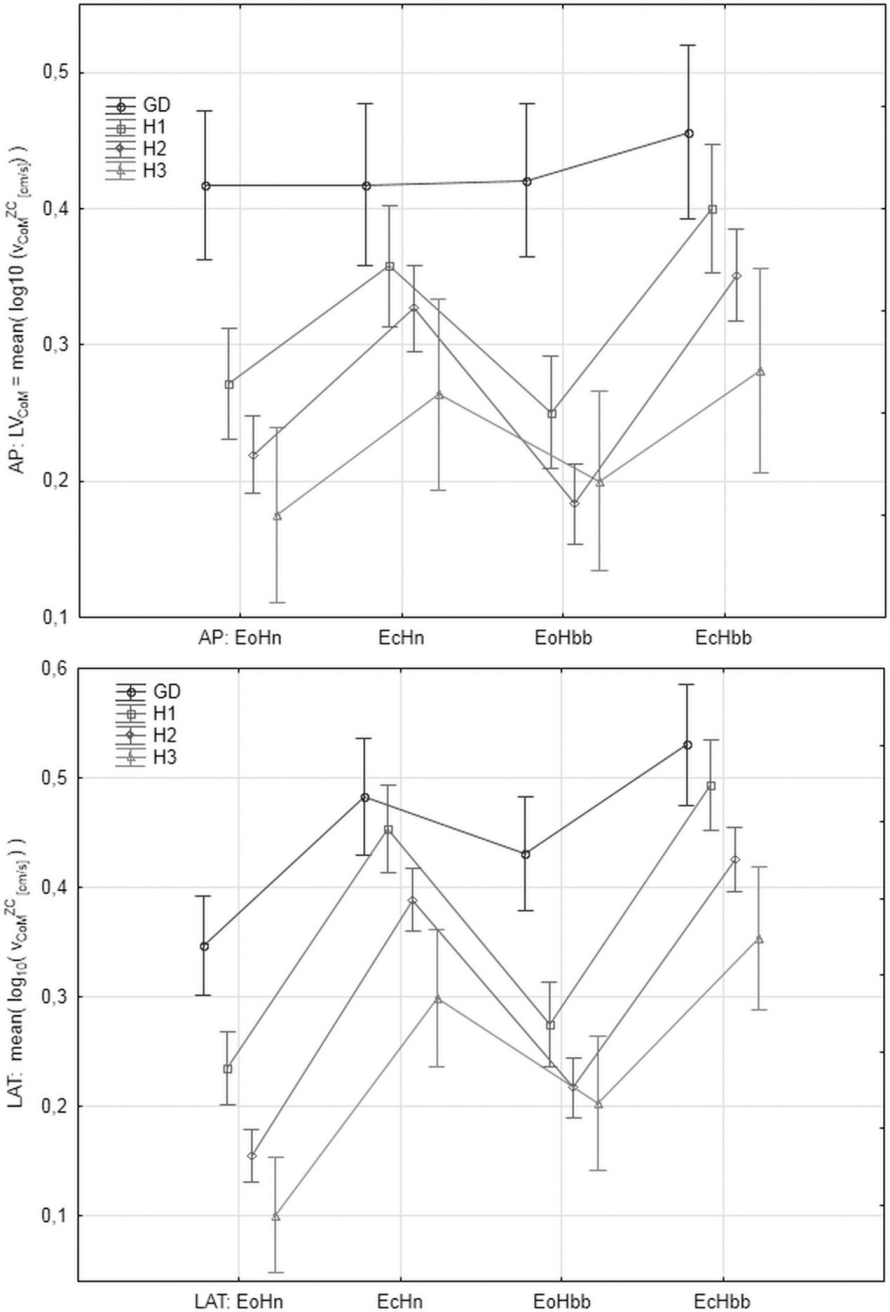
The mean values of *LV*_*CoM*_ = mean(log_10_(*v_CoM_^ZC^*)) in the subsequent patients and registration condition groups. *v*_*CoM*_^*ZC*^ represents the velocity of *x*_*CoM*_ signal in the given Zero-Crossing point. Upper: antero-posterior (AP), bottom: lateral (LAT) component of the signal. Markers denote the standard error of the mean.

**Fig 10:**
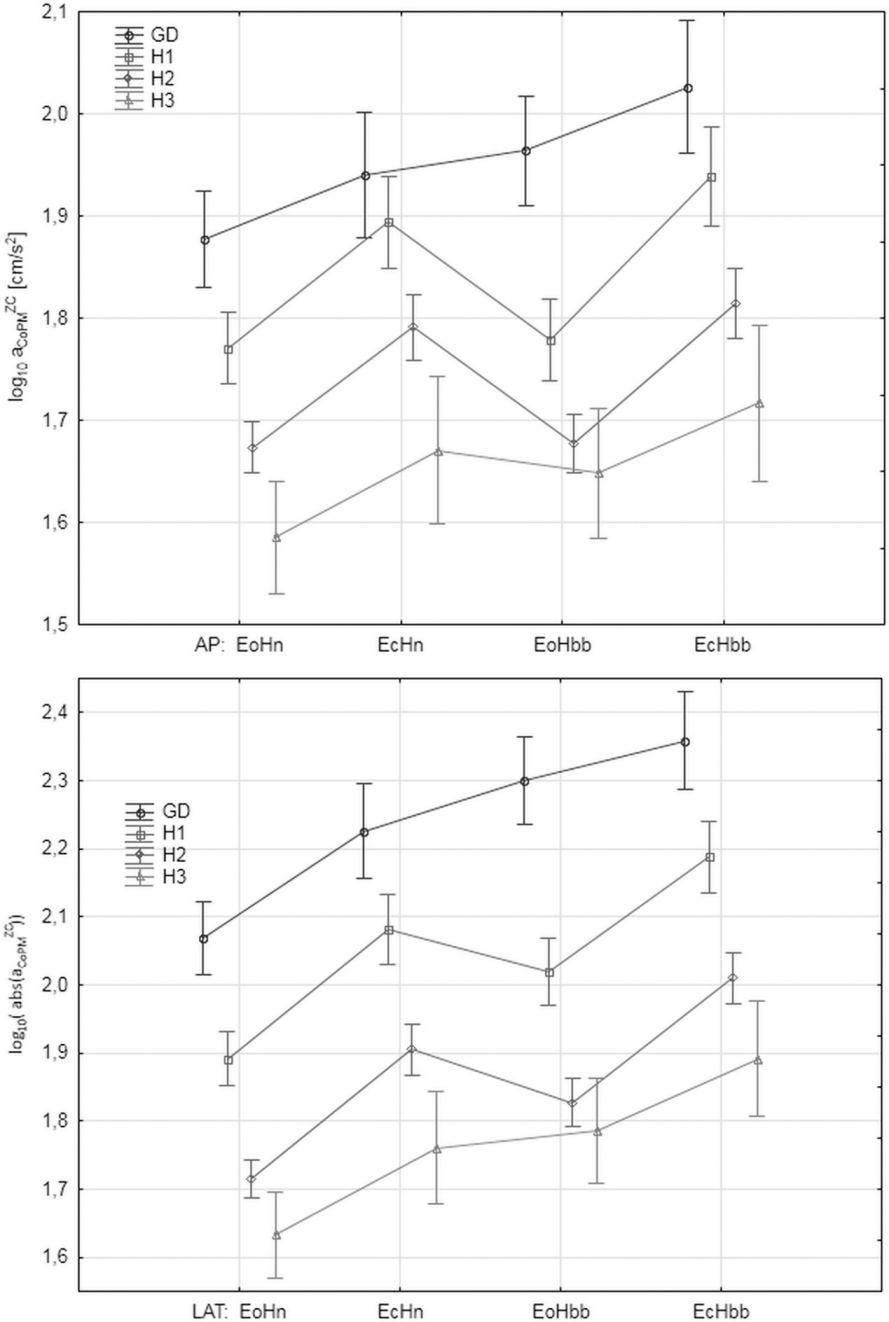
The mean values of *LA*_*CoPM*_ = mean(log_10_(*a_CoPM_^ZC^*)) in the subsequent patients and registration condition groups. ***a***_***CoPM***_^***ZC***^ **represents the acceleration of the *x***_***CoPM***_ **signal in the given Zero-Crossings point.** Upper: antero-posterior (AP), bottom: lateral (LAT) component of the signal. Markers denote the standard error of the mean.

**Fig 11:**
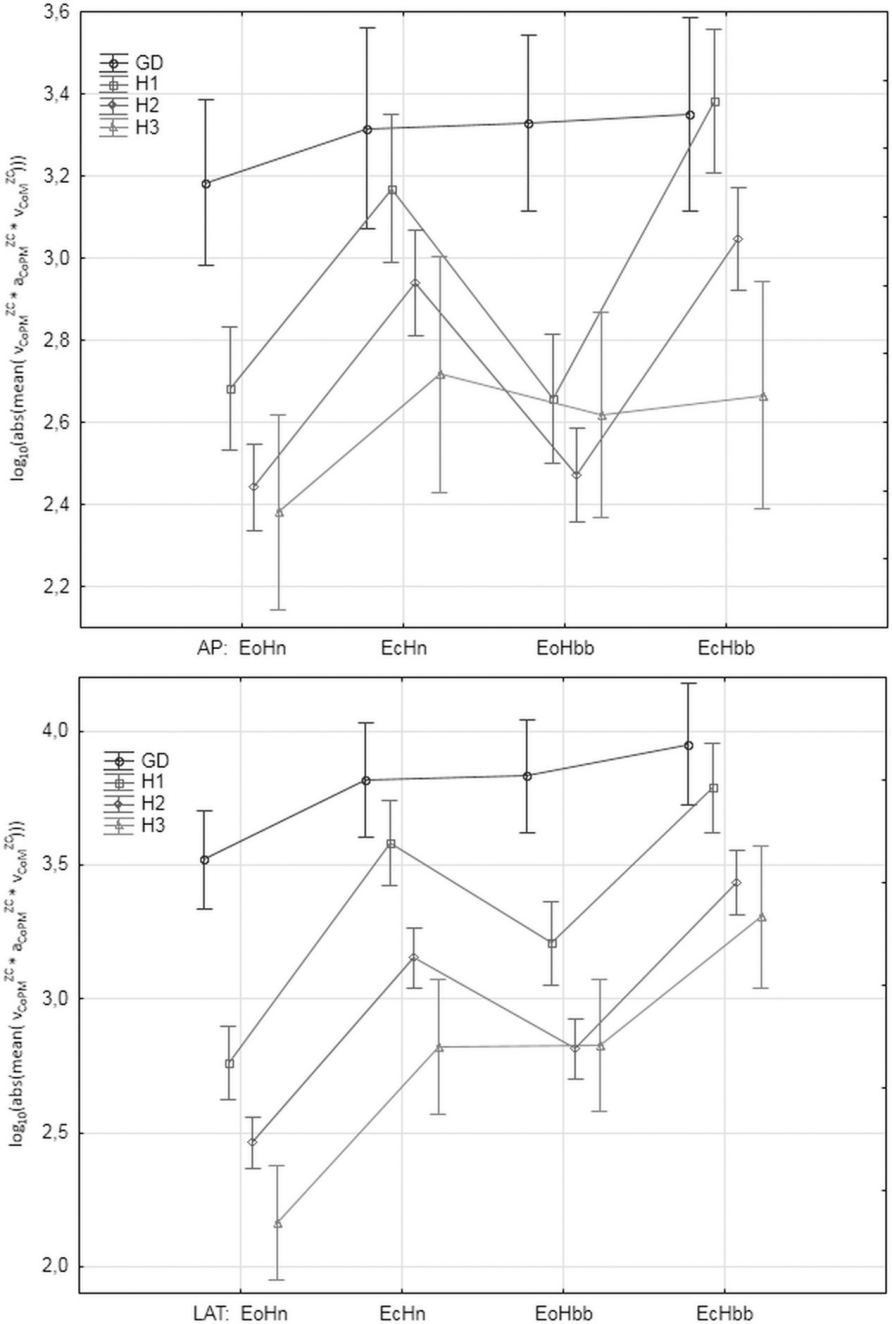
The mean values of *LM* = log_10_ (|mean(*v_CoPM_^ZC·^ v_CoM_^ZC·^ a_CoPM_^ZC^*)*|* ) in the subsequent patients and registration condition groups. *LM* represents the simple function of all the analyzed Zero-Crossing derived parameters. Upper: antero-posterior (AP), bottom: lateral (LAT) component of the signal. Markers denote the standard error of the mean.

**Fig 12:**
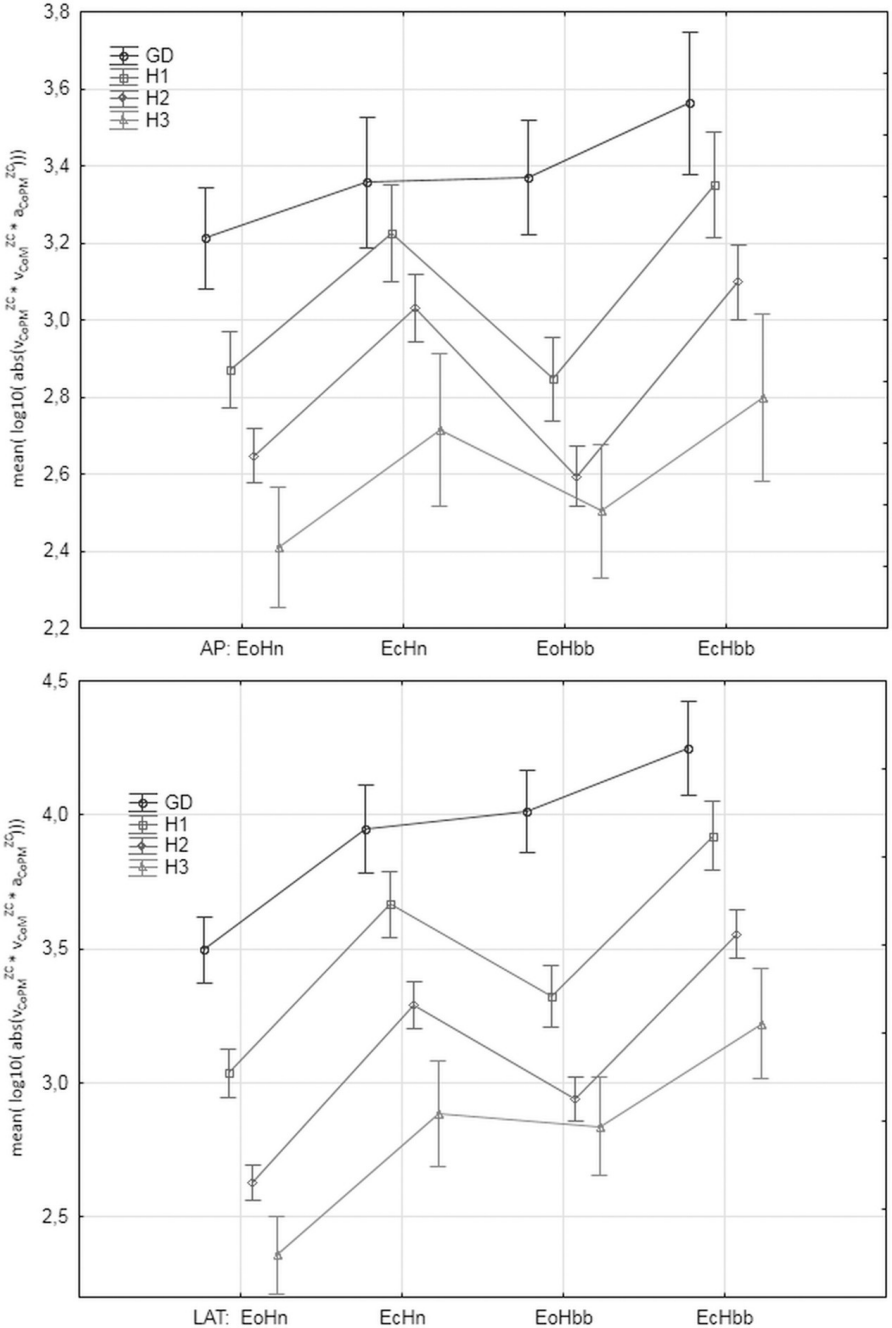
The mean values of *ML* = mean(log_10_ (|*v_CoPM_^ZC·^ v_CoM_^ZC·^ a_CoPM_^ZC^|* )) in the subsequent patients and registration condition groups. *ML* represents the simple function of all the analyzed Zero-Crossing derived parameters. Upper: antero-posterior (AP), bottom: lateral (LAT) component of the signal. Markers denote the standard error of the mean.

"Figs 8–12" show the results for the new analyzed variables: *LV*_*CoM*_, *LV*_*CoPM*_, *LA*_*CoPM*_, *ML* and *LM* being estimated and based on the Zero Crossing points of the signals. The markers in graphs denote the standard error of the mean in the analyzed groups.

Let us observe that all the figures present, in general, have similar relations between parameter values in the analyzed groups. However, the current paper focuses on the ability of the individual parameters to distinguish between analyzed groups and registration conditions rather on their absolute values. Thus, the point of interest is the width of the error markers in subsequent figures. The lower width and higher distance between error bars denotes the higher ability of the given parameter to detect the balance deterioration factor which is connected with the smaller *p* value in the t-Student test and higher Cohen's *d* value.

## Discussion

### Parameters distribution

In order to understand the reasons for the log-normal distribution of the analyzed parameters in subsequent groups let us analyze the equations of the inverted pendulum model. It is characterized by the acceleration of the falling of the body to depend linearly on the deflection of CoPM (*a*_CoM_ = *k*· *x*_CoPM_) **[4]**. When no corrective muscle impulses occur in time *t* (*x*_*CoP*_ does not move) then the *x*_*CoM*_ (and *x*_*CoPM*_) positions move away from the *x*_*CoP*_ (body falls down). The acceleration of falling increases with the increasing distance *x*_*CoPM*_. The solution for the differential equation describing this process proves that the *x*_*CoPM*_ distance grows approximately exponentially with time: *x*_*CoPM*_(*t*)~ e^*bt*^. ($b=\sqrt{k}$). This relation can be converted to the form: log(*x*_*CoPM*_) ~ *t*.

The observed distribution of the estimated logarithmic parameters in the patient and registration condition groups is approximately Gaussian, thus, it can be carefully concluded that the mean time of falling *t*, after which the corrective impulse takes place, also possesses approximately the Gaussian distribution. On the other hand, if the corrective impulse is a bit delayed then the size of the *x*_*CoPM*_ deflection grows quickly which requires a stronger corrective impulse stopping the *xCoM* to fall down and taking the *x*_*CoM*_ point back to the center of the feet point.

Another conclusion can be also drawn that the median or geometric mean could be a better estimator of the central values of the analyzed parameters. Let us recall that the geometric mean of *X* corresponds to the arithmetic mean of log(*X*).

### Effect of the decomposition of CoP into CoM and CoPM

In the first step, let us observe that the decomposition of the original (*x*_*CoP*_) signals into *x*_*CoM*_ and *x*_*CoPM*_ improves the ability to detect the deterioration factors even when using the old standard parameters *LS* and *LE*. The comparison of the statistical significances between *x*_*CoP*_ and *x*_*CoPM*_ shows that in the majority of cases the significance for *x*_*CoPM*_ is stronger than for the original *x*_*CoP*._ The significances for *x*_*CoM*_ were not higher than that for *x*_*CoP*_ and they are not presented in "Tables 1 and 2". The higher significance of *LS* and *LE* for *x*_*CoPM*_ can be explained by the fact that *x*_*CoPM*_ represents balance corrections impulses which are to a lower degree charged with the slow drift of the *x*_*CoM*_ and represents the proper activity of the human postural control system.

### Looking for the new balance deterioration parameters

The main problem in the current posturographic signal analysis research is the low ability of the, until now, analyzed parameters to serve as diagnostic parameters in neurology and orthopedics to detect different pathologies in neurological and musculoskeletal systems. The problem seems mainly to be connected with different general muscle tonus during posturographic registration in different participants which causes a different size of the swinging of the body [35–37]. In other words, some participants seem not to use all their ability to keep in an upright position which increases the swinging over the feet rectangle, however, without general balance deterioration. Thus, the main aim for future research is to look for those signal parameters which could be independent of paid attention and general participant muscle stiffness. In the current paper, some parameters being derived on the basis of Zero Crossing points have been analyzed.

The statistical analysis showed that the decomposition of the original CoP signal into CoM and CoPM components improves the ability to distinguish between different balance quality states.

Deriving the velocity and acceleration of CoPM and CoM in Zero Crossing points improves the precision of the determination of balance state quality. Especially, the velocity of CoPM in the ZC points (*LV*_*CoPM*_) for LAT direction is the most advantageous parameter. Similar results pointing to the increased lateral rather than antero-posterior sway in older adults was reported by Sparto et al. [38], as well. The variability of its mean value is relative smaller which is visible as the narrower error bars in "Fig 8", higher statistical significances of *p* of the t-Student test and higher Cohen's *d* effect size. The parameters *LV*_*CoM*_ and *LA*_*CoPM*_ for LAT direction look interesting, as well. *LA*_*CoPM*_ looks to be the best for the Age Factor. The analysis for AP direction is a bit ambiguous for general conclusions. The parameters *LM* and *ML* being simple combinations of *v*_*CoPM*_^*ZC*^, *v*_*CoM*_^*ZC*^ and *a*_*CoPM*_^*ZC*^ do not improve significantly the determination of balance quality. *ML* gives similar results to *LV*_*CoPM*_. *ML* is more significant than *LV*_*CoPM*_ for the Eyes Factor EF and less significant for Head Factor and Age Factor. *LM* seems useless, in general. Therefore, attention must be paid to the order of the 'mean' and 'log' operations when planning future experiments.

The separate problem being connected with presented analysis is the precision of ZCs extraction. Different methods of ZC extraction discussed by Lafond [24] are charged with different kinds of error. Improving the precision of ZC extraction may improve the ability of this method to distinguish between healthy and ill persons. Another parameters based on ZC points may be analyzed to possess the higher diagnostic value, as well.

## Supporting information

S1 File Supplementary material. The .zip file contains the .m matlab files used for calculating the parameters and .xlsx files with values of the subsequent analyzed parameters for each patient.
(ZIP)
